# 3′UTR of mRNA Encoding CPEB Protein Orb2 Plays an Essential Role in Intracellular Transport in Neurons

**DOI:** 10.3390/cells12131717

**Published:** 2023-06-25

**Authors:** Eugene N. Kozlov, Roman V. Deev, Elena V. Tokmatcheva, Anna Tvorogova, Zaur M. Kachaev, Rudolf A. Gilmutdinov, Mariya Zhukova, Elena V. Savvateeva-Popova, Paul Schedl, Yulii V. Shidlovskii

**Affiliations:** 1 Laboratory of Gene Expression Regulation in Development, Institute of Gene Biology, Russian Academy of Sciences, 119334 Moscow, Russia; ugin.sfu@gmail.com (E.N.K.); rde3v@yandex.ru (R.V.D.); k-z-m@mail.ru (Z.M.K.); rudolf.gilmutdinov@mail.ru (R.A.G.); zhukovamv@gmail.com (M.Z.); pschedl@princeton.edu (P.S.); 2 Institute of Physiology, Russian Academy of Sciences, 188680 St. Petersburg, Russia; tokmatcheva@mail.ru (E.V.T.); esavvateeva@mail.ru (E.V.S.-P.); 3 Center for Precision Genome Editing and Genetic Technologies for Biomedicine, Institute of Gene Biology, Russian Academy of Sciences, 119334 Moscow, Russia; annatvor@mail.ru; 4 Department of Molecular Biology, Princeton University, Princeton, NJ 08544-1014, USA; 5 Department of Biology and General Genetics, Sechenov First Moscow State Medical University (Sechenov University), 119992 Moscow, Russia

**Keywords:** CPEB proteins, 3′-untranslated region, mRNA localization, long-term memory

## Abstract

Intracellular trafficking plays a critical role in the functioning of highly polarized cells, such as neurons. Transport of mRNAs, proteins, and other molecules to synaptic terminals maintains contact between neurons and ensures the transmission of nerve impulses. Cytoplasmic polyadenylation element binding (CPEB) proteins play an essential role in long-term memory (LTM) formation by regulating local translation in synapses. Here, we show that the 3′UTR of the *Drosophila* CPEB gene *orb2* is required for targeting the *orb2* mRNA and protein to synapses and that this localization is important for LTM formation. When the *orb2* 3′UTR is deleted, the *orb2* mRNAs and proteins fail to localize in synaptic fractions, and pronounced LTM deficits arise. We found that the phenotypic effects of the *orb2* 3′UTR deletion were rescued by introducing the 3′UTR from the *orb,* another *Drosophila* CPEB gene. In contrast, the phenotypic effects of the 3′UTR deletion were not rescued by the 3′UTR from one of the *Drosophila* α-tubulin genes. Our results show that the *orb2* mRNAs must be targeted to the correct locations in neurons and that proper targeting depends upon sequences in the 3′UTR.

## 1. Introduction

Proper distribution of mRNAs and proteins within the cell plays a critical role in embryogenesis, cell differentiation, and the functioning of specialized cell types [1,2,3,4]. As is known, mRNA localization coupled with on-site translation is one of the common strategies used to localize proteins to appropriate cell compartments [5,6,7]. Neurons, which have long outgrowths, termed axons, and much shorter outgrowths, dendrites, are a context in which mRNA localization and on-site translation are particularly important. Specific mRNAs and proteins must be localized in the distal part of neuronal outgrowths (neuritis) [8,9] to ensure synaptic transduction of nerve impulses between neurons [10]. 

Cytoplasmic polyadenylation element (CPE) binding proteins (CPEBs) are a family of proteins that are known to play a key role in on-site translational regulation [11]. Depending on the context and the mRNA, CPEBs can either repress or activate translation through a polyA-dependent mechanism [12,13]. In addition to regulating translation, CPEBs can mediate the intracellular transport of mRNAs in neurons according to several studies [14,15,16].

Neuron-specific mRNAs typically have longer 3′UTRs compared with mRNAs of other cell types [17]. These long 3′UTRs have regulatory elements to target the mRNAs to specific compartments within the cell and to control their translation. Recent studies have shown that long 3′UTRs can additionally play noncanonical roles, mediating the protein–protein interactions [18], forming new intracellular domains [19], and acting independently of the mRNA-coding region [20,21].

The *orb2* gene has multiple mRNA isoforms of different lengths, including a very long one, which is expressed in nerve cells [22]. The *orb2* gene encodes one of the two *Drosophila melanogaster* CPEBs and plays an important role in the functioning of the nervous system [23,24]. There are two Orb2 protein isoforms, Orb2A and Orb2B. The shorter isoform Orb2A forms oligomers with amyloid-like properties and can induce a similar conversion of the larger Orb2B isoform [25]. Depending on its oligomer state, the Orb2 protein can control the local translation of mRNAs in synapses in response to external stimuli. As such, Orb2 plays a central role in the formation of long-term memory (LTM) [26]. Local translation in a stimulated synapse is thought to change the composition and physiological properties of receptors in the postsynaptic membrane. This results in long-term potentiation (or, much less frequently, long-term depression), that is, changes in the strength of nerve impulse transmission. At the next step, the activated synapse undergoes morphological alterations, which depend upon transcription. Together the changes form a stable memory engram [27,28,29].

The Orb2 protein has been identified as a critical participant in LTM formation in *Drosophila* [30,31]. A deletion of the Q-domain, which mediates Orb2 oligomerization, or even a single amino acid substitution in this part of the protein results in the inability to form LTM. The Orb2A isoform is expressed at very low levels in the fly brain, but its expression in dopaminergic neurons of the mushroom bodies (a center of memory acquisition and consolidation) is critical for LTM formation [26].

We have shown previously that a deletion of the 3′UTR sequences from the mRNA isoforms that encode the larger Orb2 protein disrupts LTM formation [32]. Our experiments suggest that LTM formation is disrupted because insufficient amounts of the Orb2 protein are expressed in the synaptic zone of neurons. To provide further evidence that LTM formation requires the localization of *orb2* mRNAs to the synaptic zones of neurons, we generated heterologous 3′ UTR replacements. For the control replacement, we selected the 3′UTR of the *Drosophila* α-tubulin gene (*αTub84B*) as it is not expected to be localized in a pattern like *orb2*. For the experiment, we selected the 3′UTR from the *orb* gene, which encodes the other *Drosophila* CPEB protein. Like *orb2,* its mRNA has localization signals and it also has functions in LTM formation [33,34]. 

## 2. Materials and Methods

### 2.1. Generation of Plasmid Constructs for Drosophila Transgenesis: Fly Strains

Full-length 3′UTR sequences from the alpha-tubulin (*αTub84B*) and *orb* genes were amplified from genomic DNA with primers containing BamHI and BglII restriction sites. The sequences of primers and the 3′UTRs are given in the Supplementary Materials. Next, the amplified fragments were cloned in a pOT2-attB-containing vector. The resulting plasmids were checked by sequencing. 

In the starting *orb2* 3′UTR deletion, *orb2^R^*, an attP site for recombination was substituted for the deleted 3′UTR sequences [35]. To generate the replacements, the *orb2^R^* fly strain was crossed with a phiC31-expressing strain (34772, DBSC, USA, genotype: y (1) w(*) P[y[+t7.7] = nos-phiC311]X; L (1)/CyO; TM2/TM6B, Tb (1)). Embryos obtained from the crosses were injected with a plasmid containing attB and the full-length sequences of the *αTub84B* and *orb* 3′UTRs. Flies with recombination events were selected by dsRed expression in the eyes; the dsRed marker was removed via Cre recombinase-mediated excision. Five independent flies with recombination events were used to generate homozygote stocks; the integrated fragments were sequenced. The resulting strains were designated as *orb2^tub^* and *orb2^orb^*.

The *Drosophila* fly strain 51324 (genotype: w (1118); PBacVK00027) from the *Drosophila* Bloomington Stock Center (DBSC, Bloomington, IN, USA) was additionally used in the study as a wild-type (WT) control to measure the mRNA and protein levels. It was shown previously that the 51,324 fly strain has the same ability to form memory as the standard *CantonS* strain, which is usually used in behavioral assays. The *CantonS* fly strain was used as a WT control in behavioral experiments.

### 2.2. Antibodies

The 4G8 (1:50 dilution) antibody against the Orb2 protein was used in Western blotting. The antibody was deposited in the Developmental Studies Hybridoma Bank (DSHB, Iowa, IA, USA) by P. Schedl. The DCSP-1 (ab49) antibody against the Cysteine-string protein (CSP) was deposited in the DSHB by E. Buchner and A. Hofbauer and was used in a 1:500 dilution. The 3C11 antibody against Synapsin was deposited in the DSHB by B.R.E. Klagge and colleagues and used at a 1:200 dilution. An anti-polychaetoid (PYD1) antibody was deposited in the DSHB by A.S. Fanning and used at a 1:500 dilution. An HRP-conjugated goat anti-mouse IgG antibody (Jackson ImmunoResearch, UK, catalog number 115-035-174) was used as a secondary antibody in Western blotting at 1:2000.

### 2.3. Behavioral Assay

A courtship-suppression paradigm was used to assess memory formation [32,36]. Flies were raised on standard *Drosophila* yeast–raisin medium at 25 °C and 60–70% humidity with a 12 h light–dark cycle. Males of a studied strain were collected without anesthesia 0–10 h after eclosion and kept individually in food vials until the tests. The training was performed using 5-day-old CS females fertilized 1 day before. Testing was performed at the age of 5 days. For training, naive males having no courtship experience were placed in a chamber with a fertilized female for 30 min for further assessment of either STM (short-term memory, immediately after training) or MTM (middle-term memory, 180 min. after training). For LTM formation, a male and a female were kept together for 5 h and assessed 2 days after training, using newly fertilized 5-day-old CS females. Naive males were used as a control. For each point, 20 males were tested. The time spent in courtship (orientation, following, wing vibration, licking, and attempted copulation) was recorded for 300 s. The courtship index (percentage of time spent in courtship) was calculated for each male. A courtship index (CI) was calculated as a ratio of courtship time to the entire observation time [36]. A learning index (LI) was calculated as LI = [(CIn − CIexp)/CIn] × 100%, where CIn is the average CI of two independent samples of males without courtship experience, and CIexp is the average CI of two independent samples of males after training. Statistical comparisons of behavioral data were conducted by a randomization test, by directly calculating the probability of rejection of the null hypothesis αR. A sampled randomization test with 10,000 permutations was used. The null hypothesis was rejected at αR < 0.05. A two-sided test was used to compare CIs, and a one-sided test for the comparison of LIs. In the latter case, no SEMs were required [36].

### 2.4. Fluorescent In Situ Hybridization (FISH)

To perform in situ hybridization, at least 20 brains were dissected from 4- to 5-day-old flies for each genotype used in the study. The brains were washed twice with PBS supplemented with 0.05% Tween-20 (PBSt) for 10 min and fixed with 3.5% paraformaldehyde for 20 min at room temperature. The brains were treated with 100% methanol at –20 °C for 5 min, and then methanol was replaced with PBS through a series of washes with descending vol/vol methanol/PBS solutions (70/30, 50/50, 30/70). Each rinse was combined with rotation and lasted 10 min (room temperature). After the final wash, the brains were washed with PBSt twice for 5 min and incubated in a prewarmed wash buffer (4× SSC, 35% formamide, 0.1% Tween-20) at 37 °C for 15 min. After incubation, the samples were placed in a hybridization buffer (Stellaris RNA FISH Hybridization Buffer, LGC Biosearch Technologies, Middlesex, UK, cat. No SMF-HB1-10) supplemented with FISH probes at a 1:10 dilution and incubated at 37 °C with continuous shaking. The next day, the samples were washed with a wash buffer at 37 °C for 4 h and with PBSt at room temperature for 2 h. The samples were mounted on a slide glass in the VectaShield mounting medium with DAPI (Vector Labs, Newark, CA, USA) and examined by microscopy. The FISH probes were manufactured by LGC Biosearch Technologies. A probe set consisted of twenty 20-nt probes complementary to part of the last common exon of the *orb2* gene. Each 20-nt probe was conjugated with Quasar 670 at the 3′ end. The sequences of all probes are presented in the Supplementary Materials.

### 2.5. RNA Isolation, Reverse Transcription, qPCR

To isolate RNA, the Trizol reagent was used according to the manufacturer’s recommendations (Molecular Research Center, Cincinnati, OH, USA, catalog number TR118). Twenty fly heads of each genotype were taken; the final RNA concentration was measured with an RNA assay kit (Thermo Fisher Scientific, Waltham, MA, USA, catalog number Q32852). Equal amounts of RNAs obtained from different biological samples were used to generate cDNAs. Reverse transcription was carried out with a reverse transcriptase RNAscribe RT kit (Biolabmix, Novosibirsk, Russia, catalog number R04-10) according to the manufacturer’s recommendations.

When performing quantitative PCR, each sample was used in technical triplicate. At least five independent biological replicas were used in every experiment. To assess the relative amount of the *orb2* mRNA in total fly head extracts, *gapdh2* was used as a reference gene to calculate 2^−ΔΔCt^. The 28S RNA was used as a reference when 2^−ΔΔCt^ was calculated to compare the distribution of the *orb2* mRNA and its potential mRNA targets (*act5C, csp*, and *pyd*) between the cell body and synaptic fractions of neurons. Sequences of primers used in the study are presented in the Supplementary Materials.

### 2.6. Crude Synaptosome Preparation

To study the protein distribution in nerve cells, 50 fly heads of each genotype were homogenized in 50 µL of 0.32 M sucrose buffer (5 mM HEPES, pH 7.4, 145 mM NaCl, 5 mM KCl, 2 mM CaCl_2_, 1 mM MgCl_2_, 5 mM glucose) supplemented with a protease inhibitor cocktail. Crude synaptosome preparations were obtained essentially as described in [37] with an extra centrifugation step added after collecting the first pellet. To study the RNA distribution, we collected at least 400 flies of each genotype. The flies were frozen in liquid nitrogen, vortexed, and sifted through two 800 and 380 µm sieves to separate heads from bodies. The 0.32 M sucrose homogenization buffer was additionally supplemented with a ribonuclease inhibitor (Ribolock, Thermo Fisher Scientific, Waltham, MA, USA, catalog number EO0382).

### 2.7. Semiquantitative Western Blot Analysis

To assess the distribution of the proteins of interest in *Drosophila* nervous cells, we used the strategy described in [32]. Twenty fly heads of each genotype were taken for analysis.

### 2.8. Confocal Microscopy

Stained samples of fly brains were scanned and imaged under a Leica STELLARIS 5 (Wetzlar, Germany) confocal microscope. We used a 40× oil objective with a numerical aperture of 1.3. Images with a frame size of 2048 × 2048 pixels and a z resolution of 1 µm were taken at a scan speed of 400. At least four brains were imaged for each genotype, and selected images were processed using LazX software (Leica Application Suite X, version 3.7.25997.6., Leica Microsystems, Wetzlar, Germany). Average intensity values in the marked brain regions were determined using Fiji 1.53t software.

### 2.9. Quantification and Statistical Analysis

Experimental data were statistically analyzed with GraphPad Prism 8.0.2. software. Graphs presented in the figures were plotted using the same software. Columns display the mean values; error bars display the standard deviation. Two-tailed Student’s *t*-test was used to compare the relative mRNA and protein concentrations between flies with different genotypes. Asterisks in the graphs indicate *p*-value < 0.05; ns, nonsignificant differences.

## 3. Results

### 3.1. Generation of Fly Strains with Replacements of Deleted Part of orb2 3′UTR

**Figure 1:**
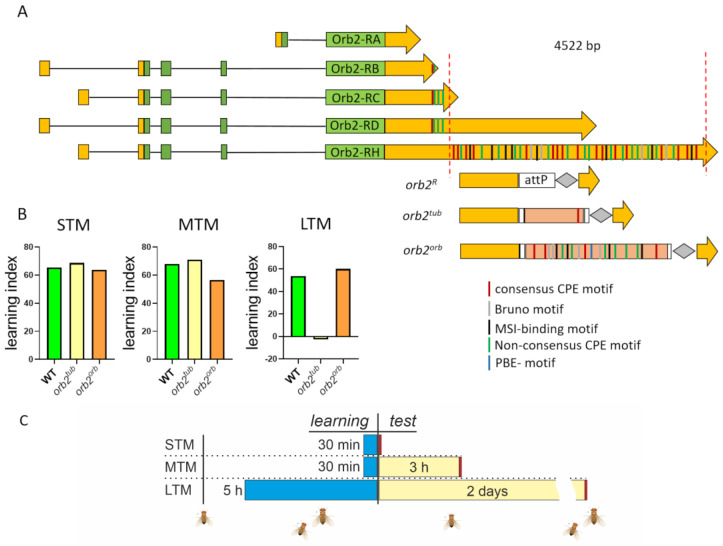
Structure of the *orb2* locus and replacements used in the study. (**A**) Structure of *orb2* transcripts. The coding regions are shown with green boxes; the 5′- and 3′UTRs, with yellow boxes. The 3′UTR part deleted in the *orb2^R^* strain is shown with red dashed lines. The structure of the modified *orb2* locus is given below. The remaining part of the 3′UTR (yellow), the attP site used for integration, the integrated sequences, and the LoxP site (gray rhombus) are also shown. Several regulatory motifs are marked with strips of different colors (see detail in text). (**B**) Learning indices calculated for short-term (STM), mid-term (MTM), and long-term (LTM) memory. STM and MTM of the *orb2^tub^* and *orb2^orb^* fly strains were the same as in the WT strain; *orb2^tub^* flies showed a significant reduction in LTM formation. (**C**) Behavioral assessment timeline. The blue bars indicate the learning time, the yellow bars indicate the time before the test, and the red bars indicate the test of the courtship behavior.

In previous studies, we generated a 4.5 kb deletion of sequences encoding two of the larger *orb2* 3′UTRs [35]. As indicated in Figure 1, the largest *orb2* 3′UTR has 37 CPE-like elements, while the *orb2^R^* deletions have only four putative CPE elements, which are located closer to the stop codon for Orb2B (hereinafter referred to as Orb2) protein. The deletion does not remove sequences from the 3′UTR sequences of the mRNA encoding the Orb2A isoform, nor does it remove sequences from the shortest *orb2B* mRNA, *orb2-RB* (see Figure 1). Analysis of the starting *orb2^R^* deletion showed that Orb2 protein levels in the synaptic zone of neurons were reduced, while the levels in neuronal cell bodies were equivalent to those in WT. We also found that the formation of long-term memory (LTM) in the *orb2^R^* mutant was significantly impaired [32].

To provide further evidence that the proper localization/regulation of *orb2* mRNA is critical for LTM formation, we replaced the deletion with two different UTR sequences, the *αTub84B* tubulin gene 3′UTR, which contains a single consensus CPE sequence, and the 3′UTR that belongs to the *orb*, another CPEB gene, and contains eight CPEs [34,38]. The full sequences are presented in Materials and Methods. We chose these two test UTRs because in the *orb2^R^* deletion, the remaining UTR sequences (see Figure 1A) had only four CPE-like elements and we supposed that among other things, the number of CPE sequences likely played a critical role in the localization of the *orb2* mRNA and/or its efficient translation. In addition, other regulatory motifs were found in the 3′UTR of the *orb2* mRNA, namely, the bird-box motif, Bruno motifs, and Musashi binding elements (MSI) [39,40]. All of them were also present in the *orb* 3′UTR. A Pumilio binding motif (PBE) is additionally found in the 3′UTR of the *orb* mRNA [41].

After phiC31-mediated integration of the UTR sequences in the attP site, the dsRed marker was removed. The final fly strains used in further experiments were designated as *orb2^tub^* and *orb2^orb^* (Figure 1B).

### 3.2. Introduction of Different Sequences in orb2^R^ Allele Does Not Affect Short-Term and Mid-Term Memory Formation but Affects Long-Term Memory

The CIs of *orb2^tub^* and *orb2^orb^* adult males were assessed to evaluate their ability to form short-term (STM) and mid-term memory (MTM) (Figure S1). Figure 1B summarizes the calculated LIs. As expected, the CIs of *orb2^tub^* flies were similar to the CIs of WT flies. The same picture was observed in *orb2^orb^* flies—i.e., their STM and MTM formation was normal (Figure 1B). Thus, we found that different sequences of the 3′UTR of the *orb2* mRNA did not affect the STM and MTM formation, as has been shown for the initial *orb2^R^* fly strain.

Next, we studied how different 3′UTR sequences affect the LTM formation when added to the *orb2*-coding region. The *αTub84B* 3′UTR did not restore the LTM formation, and *orb2^tub^* flies showed the same CIs as naive males 2 days after training. In contrast, *orb2^orb^* flies showed a significant reduction in CI just after training and retained this reduction 2 days after training. Thus, a replacement of the 3′UTR sequence deleted from the *orb2* gene with the *orb* 3′UTR sequence leads to complete recovery of the LTM formation.

### 3.3. Different 3′UTR Sequences Used to Replace Deletion of orb2 3′UTR Do Not Influence mRNA Level but Affect Protein Level of Orb2 in Total Fly Head Extracts

**Figure 2:**
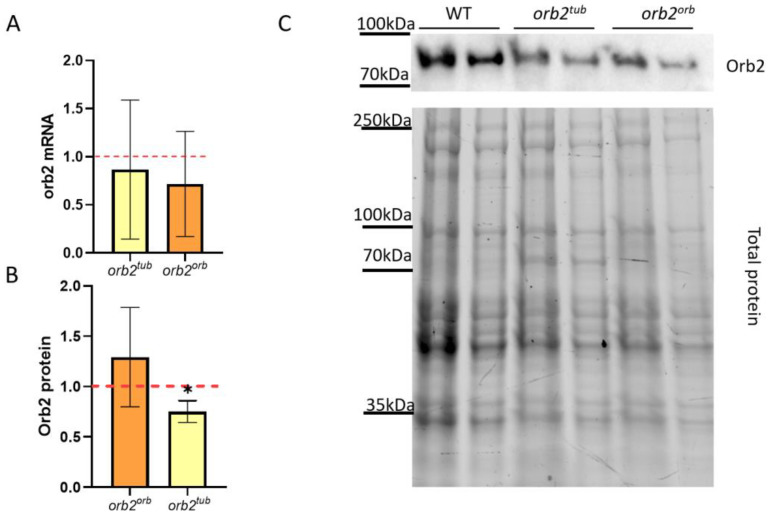
Effects of different sequences inserted in the *orb2* 3′UTR on *orb2* gene expression. (**A**) Relative levels of the *orb2* mRNA detected in heads of *orb2^tub^* and *orb2^orb^* flies by qPCR. A red dashed line presents the *orb2* mRNA level in WT flies, which was used for normalization. Columns show the mean mRNA levels detected in *orb2^tub^* and *orb2^orb^* (*t*-test, *n* = 6, SD is shown). (**B**) Relative levels of the Orb2 protein in total head extracts from *orb2^tub^* and *orb2^orb^* flies. A red dashed line presents the Orb2 protein level in WT flies, which was used for normalization. The mean Orb2 protein level was found to decrease in *orb2^tub^* flies (*n* = 6, SD is shown, asterisk shows *p* < 0.05), while *orb2^orb^* flies had the same Orb2 protein level as WT flies. (**C**) Western blot analysis of head lysates from *orb2^tub^* and *orb2^orb^* flies with antibodies against the Orb2 protein (**top**). Total protein staining (**bottom**) was used for normalization in a twofold serial dilution.

To better understand why LTM formation in *orb2^tub^* and *orb2^orb^* males is different, we assessed how insertions into the *orb2^R^* allele affect the *orb2* mRNA level. The relative *orb2* mRNA level mRNA was calculated by the ΔΔCt method with *gapdh2* used as a reference gene. The results are presented in Figure 2A. We found that the *orb2* mRNA level did not differ from the WT level in both the *orb2^tub^* and *orb2^orb^* fly strains. The same result was observed in the *orb2^R^* strain [32]. Thus, the insertion of an additional sequence in place of the deleted part of the *orb2* gene does not change the relative production or the stability of the *orb2* mRNA.

**Figure 3:**
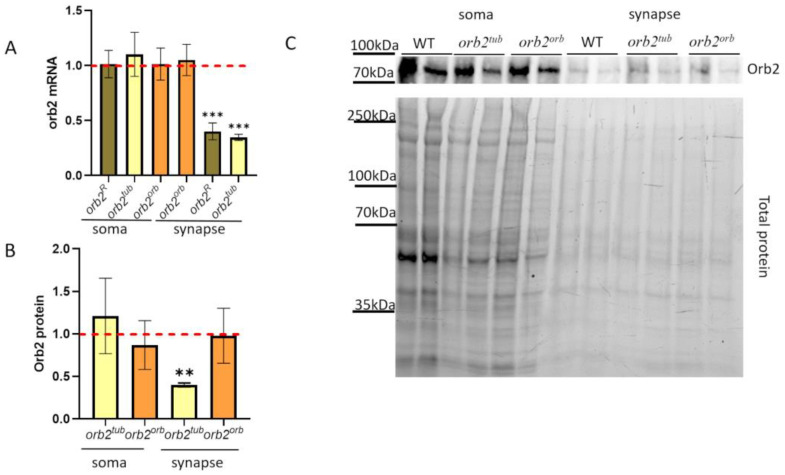
Subcellular distribution of the *orb2* mRNA and protein in neurons. (**A**) Relative levels of the *orb2* mRNA were detected in fractions of neurons of the *orb2^R^*, *orb2^tub^*, and *orb2^orb^* fly strains by qPCR. A red dashed line presents the *orb2* mRNA level in WT flies, which was used for normalization. Columns show the mean mRNA levels detected in *orb2^R^*, *orb2^tub^*, and *orb2^orb^* (*t*-test, *n* = 8, SD is shown). Asterisks show significant changes (*p* < 0.001) in the *orb2* mRNA level in the synaptic fractions of the *orb2^R^* and *orb2^tub^* strains. (**B**) Relative mean levels of the Orb2 protein in the soma and synaptic fractions from *orb2^tub^* and *orb2^orb^* flies. A red dashed line presents the Orb2 protein level in WT flies, which was used for normalization. Flies of the *orb2^tub^* strain showed a decrease in Orb2 protein amount (*n* = 6, SD is shown, asterisks show *p*-value < 0.01), while *orb2^orb^* flies had the same Orb2 protein level as WT flies. (**C**) The top panel shows an example of staining the fractions from different fly strains with anti-Orb2 antibodies. Each sample was examined in a twofold dilution as well. The bottom panel shows the total protein.

Next, the Orb2 protein level was assessed in total fly head extracts of the *orb2^tub^* and *orb2^orb^* fly strains. Chemiluminescent signals from staining with antibodies against the Orb2 protein were normalized to the total protein in the sample. We found that *orb2^orb^* flies had the same Orb2 protein level as WT flies. In contrast, *orb2^tub^* flies showed a 34% decrease in Orb2 protein level as compared with WT flies (Figure 3B). The results demonstrate that the *orb* 3′UTR sequence restored the total Orb2 protein amount in the fly brain, while the tubulin gene 3′UTR did not elevate the Orb2 protein amount to the WT level. The *orb2^tub^* and initial *orb2^R^* strains showed a similar level of decreasing of Orb2 protein amount.

### 3.4. 3′UTR of orb2 Gene Is Essential for Intracellular Transport of orb2 mRNA

The next question to study was how the sequences introduced into the *orb2* 3′UTR influence the *orb2* mRNA intracellular distribution. We applied the previously used biochemical approach to separate the soma and synaptic regions [32]. The results are presented in Figure 3A. We did not find any difference in the *orb2* mRNA level from the WT in the soma fraction of neurons in both the *orb2^tub^* and the *orb2^orb^* fly strains. The synaptic fraction of the *orb2^orb^* strain was similar to the WT in the amount of the *orb2* mRNA. In contrast, the *orb2^tub^* synaptic fraction displayed a nearly threefold reduction in *orb2* mRNA compared with the WT. We checked the distribution of the *orb2* mRNA in the initial *orb2^R^* fly train. The amount of the *orb2* mRNA in the soma of neurons was found to be the same as in the WT, whereas a decrease in the *orb2* mRNA level was observed in the synaptic fraction, similar to the *orb2^tub^* strain. Thus, we can conclude that the *orb2* 3′UTR sequence is essential for localizing the *orb2* mRNA to the synaptic compartment of the nerve cell.

### 3.5. 3′UTR of orb2 Gene Is Essential for Orb2 Protein Distribution

To analyze the Orb2 protein distribution in neurons of the *orb2^tub^* and *orb2^orb^* fly strains, we prepared the fractions of cell bodies and synaptosomes. We found that the Orb2 protein amount in the cell bodies of the strains did not differ from that in WT flies. In the synaptosome fraction, the Orb2 protein amount in *orb2^orb^* flies did not differ from that in WT flies, whereas *orb2^tub^* flies showed a more than twofold reduction in the Orb2 amount (Figure 3A). Thus, the distribution of the Orb2 protein among the fractions in *orb2^tub^* flies was similar to that reported previously for *orb2^R^* flies [32]. The results have led us to the conclusion that the 3′UTR of the *orb2* gene is essential for the proper localization of the *orb2* mRNA and protein to synapses.

### 3.6. Sequence in 3′UTR of orb2 Is Important for mRNA Localization to Neuron Outgrowths

**Figure 4:**
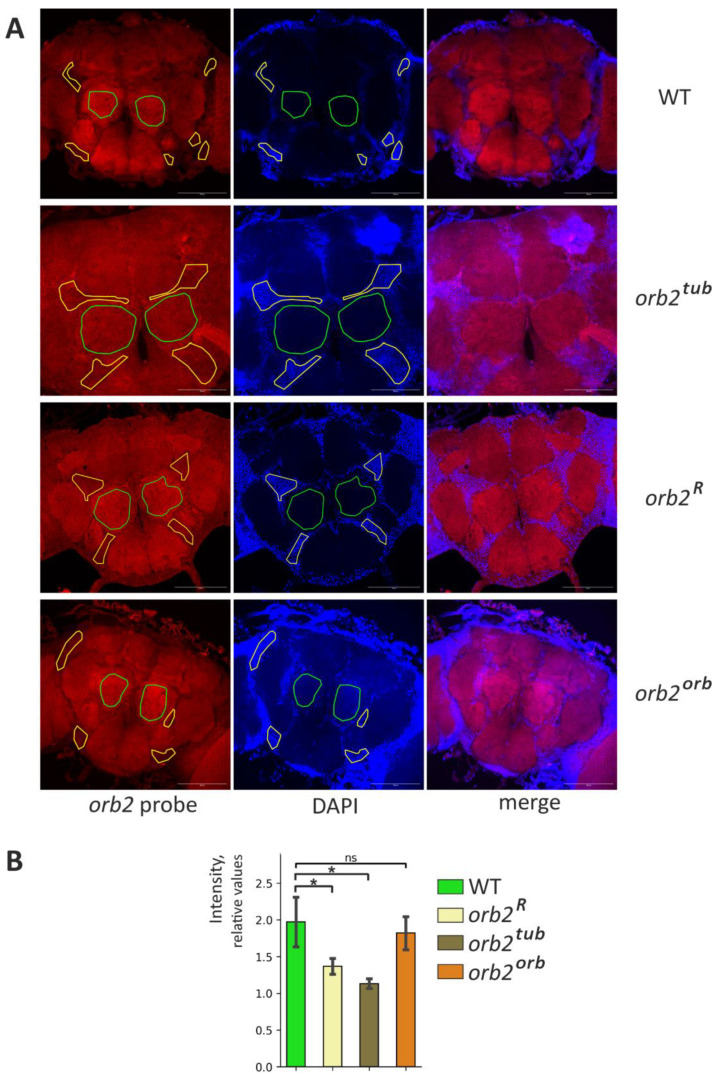
Distribution of *orb2* mRNA in the fly brain. (**A**) Staining of whole-mount brain samples with anti-*orb2* probes (red channel) and staining of nuclei with DAPI (blue channel). The same exposure was used to take images in the cases of the WT and *orb2* mutants. The neuropile regions are marked with a green line. The areas marked by the yellow line are the neuronal body regions. Scale bar, 100 µm. (**B**) The mean intensity of the neuropile regions relative to the neuronal nucleus regions in the indicated fly lines (Mann-Whitney test, *n* = 4, SD is shown, asterisk shows *p*-value < 0.05, ns is nonsignificant). The relative intensity of neuropile staining is reduced in the *orb2^R^* and *orb2^tub^* preparations.

To more precisely study the mRNA distribution in the fly brain for different *orb2* alleles, in situ hybridization with Stellaris probes was performed using whole-mount brain samples. The *orb2* mRNA was abundantly present in neuropile regions in WT flies (Figure 4A, Figures S2 and S3). We also found the *orb2* mRNA in the soma of neurons, as has been shown before [23]. When studying *orb2^R^* fly brains, we found that staining was decreased in neuropile regions and that the signal from the *orb2* probe was distributed evenly in different parts of the brain as compared with the WT (Figure 4A, Figures S2 and S3). We quantified the signal intensities of the *orb2* mRNA in the neuropile and soma regions and then calculated their ratio. The relative intensity of the *orb2* mRNA in the neuropile regions is significantly reduced in *orb2^R^* mutants (Figure 4B).

A hybridization pattern similar to the *orb2^R^* pattern was observed in *orb2^tub^* samples. Regions enriched in axons were poorly stained with the *orb2* probe (Figure 4A, Figures S2 and S3). In contrast, a recovery of the *orb2* mRNA distribution observed in WT brains was detected in *orb2^orb^* brains. This is confirmed by a quantitative assessment of staining intensity (Figure 4B).

An interesting feature of the *orb2* mRNA distribution was additionally observed in the neuron soma. A perinuclear ring with several bright speckles per cell was formed in the region of the calyx (dendritic input for Kenyon cells) by the *orb2* mRNA (Figures S4 and S5). The speckles were clearly observed in WT flies. Sites of a high local concentration of the *orb2* mRNA in the soma may be assumed to act as sites of RNP assembly. The same picture was found in *orb2^orb^* brains. In *orb2^R^* and *orb2^tub^* flies, the perinuclear ring was preserved, but the speckles were hardly distinct from other *orb2* mRNA fractions localized in the soma. In addition, the *orb2* mRNA was distributed more evenly between the cell soma and the calyx in *orb2^R^* and *orb2^tub^* brains and the background staining was stronger compared with the WT.

**Figure 5:**
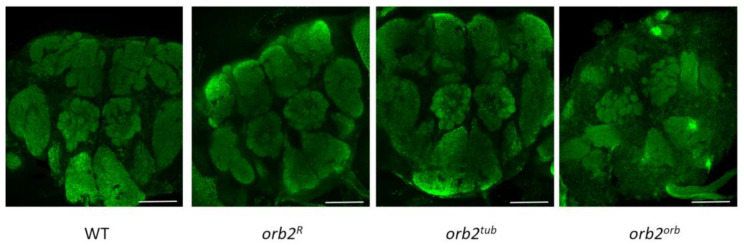
The brain structure of the studied mutants. Staining with antibodies against synapsin. The brain structure in the strains *orb2^R^*, *orb2^tub^*, and *orb2^orb^* is indistinguishable from that in WT brains. Scale bar, 50 µm.

The changes in *orb2* mRNA distribution observed in *orb2^R^* and *orb2^tub^* could be attributed to changes in brain structures. To check this assumption, brain samples were stained with antibodies against Synapsin, which is a marker of neuropile regions. The results are presented in Figure 5. We did not find any obvious change in brain organization of the *orb2^R^*, *orb2^tub^,* and *orb2^orb^* strains compared with the WT.

### 3.7. Distribution of mRNAs and Proteins of Potential Orb2 Targets in Nerve Cells

We have previously found that the Csp and Pyd proteins, whose mRNAs are potential targets of the Orb2 protein, elevate their representation in synapses of *orb2^R^* flies. *csp* encodes a chaperone associated with synaptic vesicles, and *pyd* encodes a protein involved in cell adhesion, which is important for mushroom body formation. The mRNAs encoding the proteins are thought to be regulatory targets of Orb2. One explanation for the increased amount of Csp and Pyd proteins in the synaptic fraction is that Csp and Pyd mRNAs are transported more efficiently and/or stabilized in the *orb2^R^* deletion mutant, resulting in increased total mRNA levels encoding these two proteins. Alternatively, both mRNAs may be subject to translational repression by Orb2. In this case, a decrease in the level of Orb2 in the synapse would result in an increase in the translation of *csp* and *pyd* mRNA.

**Figure 6:**
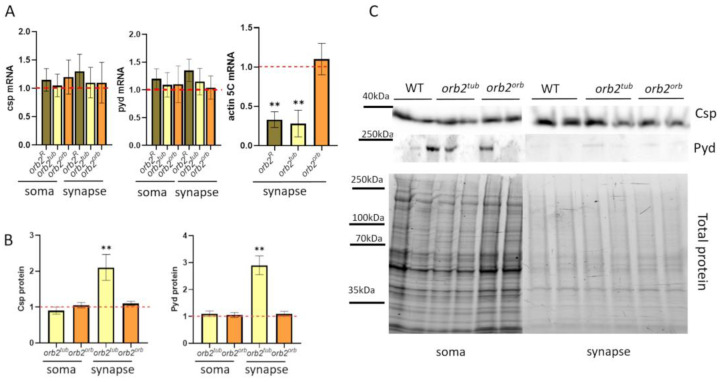
Distribution of potential Orb2 targets in nerve cells. (**A**) Relative mean levels of the *csp* and *pyd* mRNAs in the soma and synaptic fractions of the fly strains. A red dashed line presents the mRNA level in WT flies, which was used for normalization. Error bars show SD; there was no significant change in mRNA levels compared with the WT. Relative mean levels of actin5C mRNA in a synaptic fraction of studied strains (*n* = 10, Student *t*-test, asterisks show *p*-value < 0.01). (**B**) Relative mean levels of the Csp and Pyd proteins in the soma and synaptic fractions in the indicated strains (*n* = 10, Student’s *t*-test, asterisks show that *p*-value < 0.01). A red dashed line presents the protein level in WT flies, which was used for normalization. (**C**) Example of staining with antibodies against the Csp and Pyd proteins (**top**) and total protein for normalization (**bottom**). Nerve cell fractions were obtained from WT, *orb2^tub^,* and *orb2^orb^* brain samples.

To explore these possibilities, we used qPCR to measure the amount of *csp* and *pyd* mRNA in soma and synapse fractions in the head obtained from *orb2^R^*, *orb2^tub,^* and *orb2^orb^* flies. As shown in Figure 6A, the relative level and distribution in the soma and synaptic fractions were the same as in WT.

Next, we examined the distribution of Csp and Pyd proteins in the soma and synaptic fractions. In the *orb2^orb^* sample, the levels of the proteins were the same as in the WT flies. However, in *orb2^tub^* samples, the levels of the proteins were elevated in the synaptic fraction compared with the WT (Figure 6B). Similar results have been obtained previously for the *orb2^R^* deletion [32].

Changes in distribution were also explored for the actin5C mRNA in the fly strains because the mRNA has been shown to provide a potential target for the Orb2 protein [42,43]. We found that its amount significantly decreased (almost three times as compared with WT flies) in the synaptic fraction in the *orb2^tub^* strain and was almost the same as in WT flies in the *orb2^orb^* fly strain (Figure 6A).

## 4. Discussion

One of the most important discoveries in neurosciences is that the synapse, rather than a single neuron, is an elementary unit for memory formation [44]. Presynaptic and postsynaptic termini contain unique sets of molecules for the transmission and reception of nerve impulses. A mechanism to target key proteins to the pre- and postsynaptic termini relies on mRNA transport followed by local translation [7,9]. Perhaps for this reason, many neuronal mRNAs have extended 3′UTRs compared with mRNAs encoding the same proteins in non-neuronal tissues [45].

Previously, we have reported the isolation and characterization of a large deletion (4.5 kb) that removes most of the sequence for two large *orb2* 3′UTRs [32]. The longest of the *orb2* 3′UTRs (*orb2-RH*) has 37 canonical and noncanonical CPE motifs, and all but 4 of these are preserved in flies with our *orb2^R^* deletion. The deletion does not appear to impact the translation of the *orb2* mRNAs in the neuronal cell body, and the levels of the Orb2 protein in this cell compartment are similar to that in the WT. On the other hand, the deletion reduces the level of the *orb2* mRNA in synaptic fractions by more than half. A concomitant reduction in Orb2 protein in the synaptic fraction accompanies this reduction in mRNA.

In the studies reported here, we used a 3′UTR replacement strategy to rescue the *orb2^R^* 3′UTR deletion. We tested the 3′UTRs derived from two different fly genes, the *αTub84B* tubulin gene and the *orb* gene for the other Drosophila CPEB protein. The former has a single CPE, while the latter has eight canonical CPEs. The *αTub84B* 3′UTR does not rescue the localization of the *orb2* mRNA to the synaptic fractions. Moreover, like in the starting *orb2^R^* deletion mutant, Orb2 protein levels in the synaptic fraction are reduced, while the protein amount in the neuronal cell body is similar to the WT. Consistent with the idea that localization of the *orb2* mRNA to synaptic fractions is important for the *orb2* function, *orb2^tub^* flies exhibit phenotypes similar to those observed for *orb2^R^*. These include elevated levels of the Csp and Pyd proteins in the synaptic fraction and pronounced deficits in LTM formation. The suggestion is supported by our analysis of the *orb* 3′UTR replacement of *orb2^orb^*. This *orb* 3′UTR replacement appears to fully rescue the phenotype of the *orb2* 3′UTR deletion. Both *orb2* mRNA and protein levels in the synaptic fraction are equivalent to the WT, as are the levels of the Csp and Pyd proteins. In addition, there is no obvious deficit in LTM formation.

Orb is known to be essential for LTM formation [33]. It is also known that the *orb* 3′UTR is essential for the proper localization of the Orb protein in the oocyte [38] through an autoregulatory loop. Additionally, the *orb* 3′UTR contains the BRE, MSI, and PBE motifs, as well as binding sites for the Pumilio protein, which could be essential for proper translation activation [39,40,41]. Fragments of similarity between the *orb* and *orb2* 3′UTRs are not long enough, but all of them contain multiple CPE sequences. Based on this observation, we assume that the number of CPE sequences in the *orb2* 3′UTR together with other *cis*-regulatory elements can somehow influence the Orb2 protein amount in synapses.

A model that could explain our results is as follows: The UTR sequences remaining in *orb2^R^* transcripts lack signals critical for neuronal transport, and the levels of the *orb2* mRNA in the synaptic fractions are consequently reduced. The same would be true for the *orb2^tub^* replacement. The levels of the Orb2 protein are reduced in both *orb2^R^* and *orb2^tub^*, and this reduction is responsible for elevated translation of the *csp* and *pyd* mRNAs in the synaptic fraction. One would suppose that the failure to properly regulate translation in the synaptic fraction is responsible for deficits in LTM formation as well. In the case of Csp and Pyd, the protein levels increase rather than decrease. Thus, Orb2 appears to repress, rather than activate, the translation of the mRNAs encoding these two proteins. Since both mRNAs have CPEs and bind with the Orb2 protein in tissue culture cells [43], it is quite possible that Orb2 regulates their translation directly. While CPEBs are known to activate translation by promoting polyadenylation, there are contexts and mRNAs whose translation is negatively regulated by CPEBs. It will clearly be of interest to determine whether the translation of other potential Orb2 targets in neurons is repressed rather than activated.

While the distribution of the *csp* and *pyd* mRNAs in *orb2^R^* and *orb2^tub^* cell bodies and synaptic fractions is similar to the WT, this is not true for the actin5C mRNA. The mRNA level of the actin5C mRNA is significantly reduced in the synaptic fraction as are the levels of the *orb2* mRNAs in *orb2^R^* and *orb2^tub^* flies. This finding raises the possibility that Orb2 may be involved in the transport of a subset of neuronal mRNAs. It will be of interest to examine the distribution of other neuronal mRNAs in *orb2^R^* and *orb2^tub^*.

**Figure 7:**
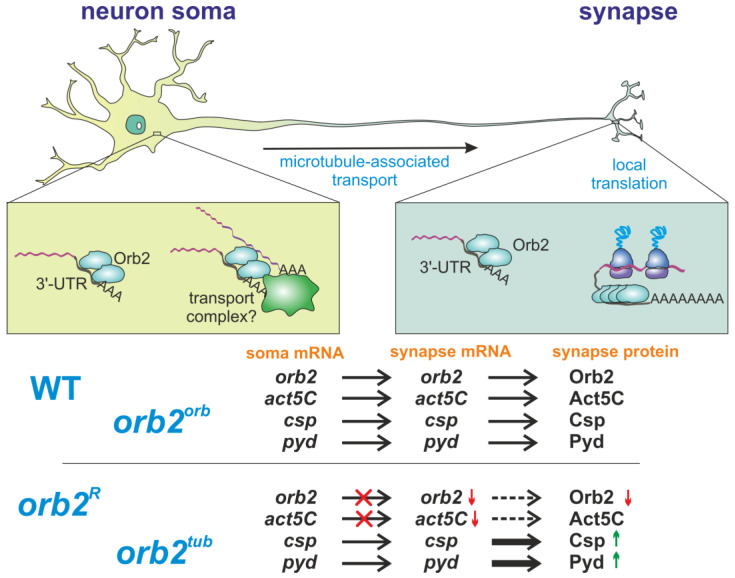
A model of the *orb2* role in neurons. The Orb2 protein participates in two major processes, intracellular transport in neurons and local translation in synapses. In the soma, Orb2 binds to the 3′UTRs of its target transcripts and participates in the formation of transport-competent mRNP. This mechanism is valid for several transcripts—e.g., Orb2 is required for intracellular transport of its own mRNA and the *act5C* mRNA to synapses, but not for transport of the *csp* and *pyd* transcripts. The *orb2^R^* and *orb2^tub^* mutants thus show lower synaptic levels of the *orb2* mRNA and Orb2 protein. In synapses, Orb2 participates in regulating translation. The *orb2^R^* and *orb2^tub^* mutants consequently show higher levels of *csp* and *pyd* mRNA translation in synapses. The *orb2^orb^* mutant shows complete rescue of the described molecular defects.

Thus, our results allow us to assume a new function for the 3′UTR of the *orb2* mRNA. In Figure 7, we summarize our hypothesis. The 3′UTR of the *orb2* mRNA mediates the interaction between Orb2 proteins in the soma of a neuron. Multiple copies of connected Orb2 proteins form an RNP complex, which can more efficiently bind to transport proteins and be transported to synapses. Once in the synapse, the Orb2 protein regulates the local translation of its target mRNAs. We suppose that further studies will provide new insights into the roles of long 3′UTRs in neuronal cell functions and their participation in intracellular transport.

## Data Availability

Not applicable.

## Supplementary Materials

The following supporting information can be downloaded at: https://www.mdpi.com/article/10.3390/cells12131717/s1, Figure S1: Courtship indices of the studied fly lines; Figures S2 and S3: Brain preparations of the WT, *orb2^R^, orb2^tub^*, and *orb2^orb^* lines; Figures S4 and S5: Brain preparations of the WT, *orb2^R^, orb2^tub^*, and *orb2^orb^* lines.

## Abbreviations

3′UTR, 3′-untranslated region; CPE, cytoplasmic polyadenylation element; CPEB, cytoplasmic polyadenylation element binding protein; STM, short-term memory; MTM, mid-term memory; LTM, long-term memory; CI, courtship index; LI, learning index.
