# Supplementary material for: 3′UTR of mRNA Encoding CPEB Protein Orb2 Plays an Essential Role in Intracellular Transport in Neurons
Source: Cells. 2023 Jun 25;12(13):1717. doi: 10.3390/cells12131717 (PMC10340461; doi:10.3390/cells12131717)
Supplement: Supplementary file 1 [file cells-12-01717-s001.zip › cells-2401781-supplementary.pdf]

Supplement for the paper

3’UTR of mRNA encoding CPEB protein Orb2 plays an essential role in intracellular transport in neurons

by Eugene N. Kozlov, Roman V. Deev, Elena V. Tokmatcheva, Anna Tvorogova, Zaur M. Kachaev, Rudolf A. Gilmutdinov, Mariya Zhukova, Elena V. Savvateeva-Popova, Paul Schedl and Yulii V. Shidlovskii

Supplementary Figures

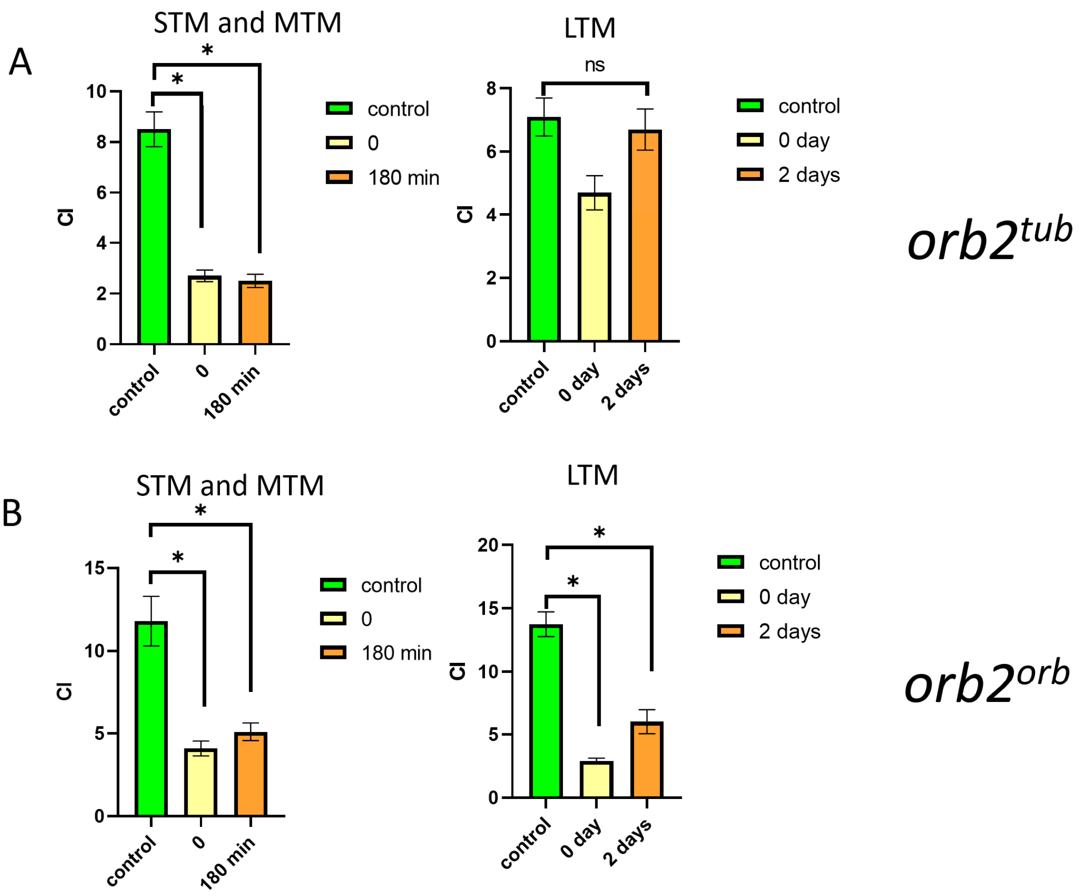

Figure S1. Courtship indices of the studied fly lines. (**A**) The left graph shows the courtship indices for the *orb2^{tub}* flies after training compared to untrained control flies. There was a significant decrease (n = 20, showing mean values and SDs as error bars) in CIs, indicating memory formation. The right graph shows the CI calculated two days after training. There was no suppression of courtship behavior; flies showed the same CI as naïve flies, thus LTM was not formed (ns, not significant). (**B**) The same values as in (**A**) are shown. The CI value is presented for the *orb2^{orb}* fly strain. We found normal formation of STM, MTM, and LTM. Asterisk shows $p < 0.05$.

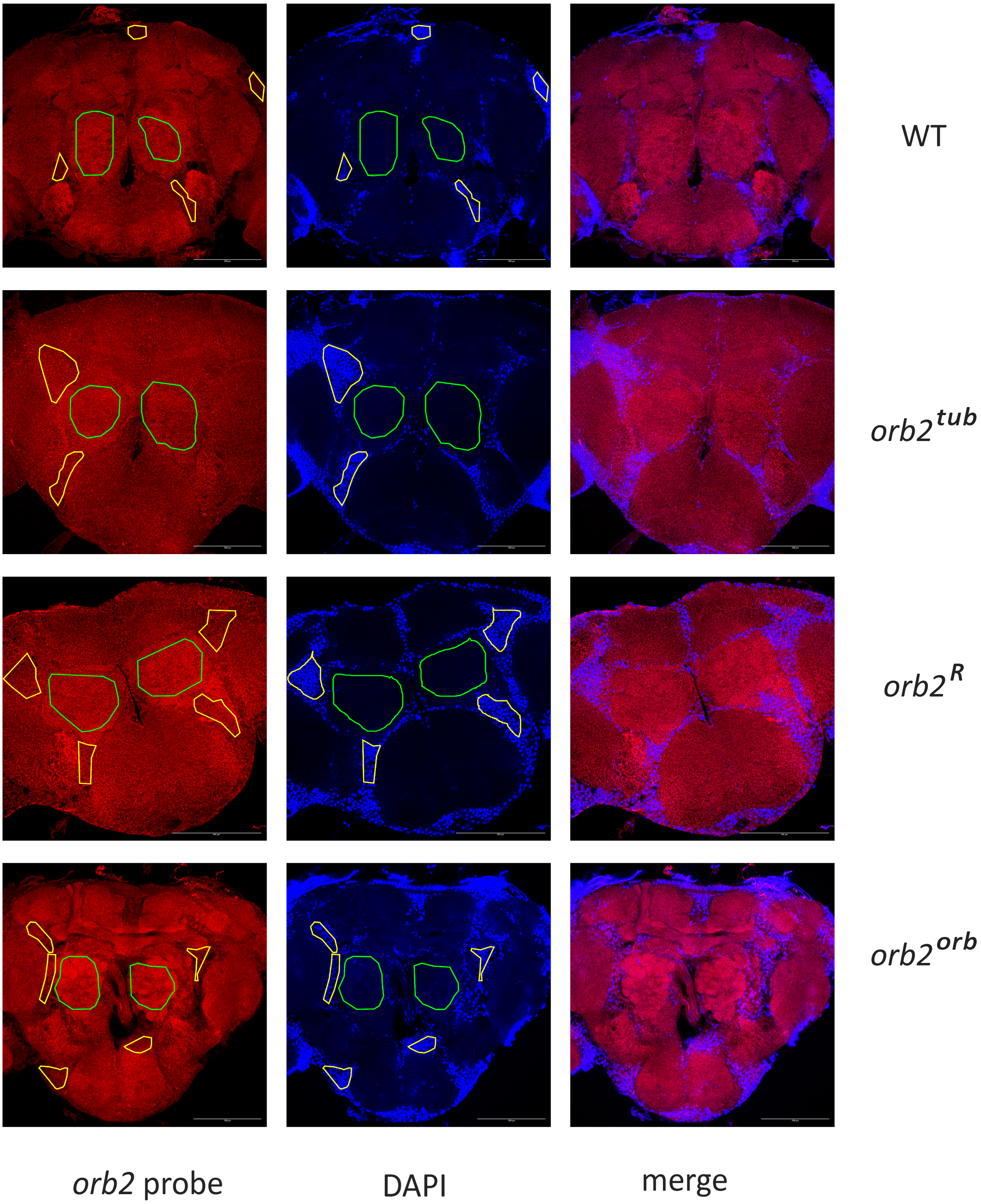

Figure S2.

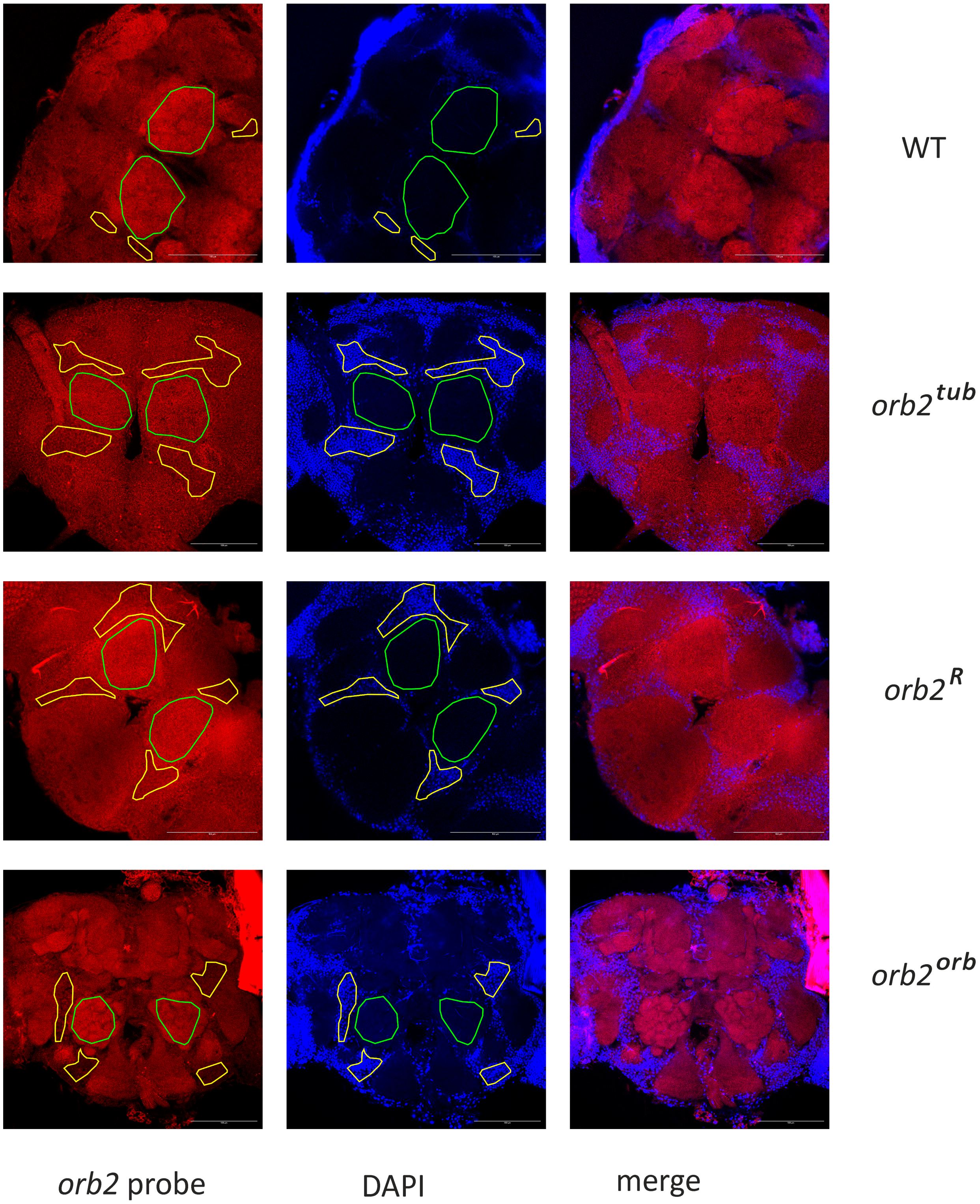

Figure S3.

Figure S3. Brain preparations of the WT, *orb2^R^*, *orb2^tub^*, and *orb2^orb^* lines. Staining of whole mount brain samples with anti-*orb2* probes (red channel) and staining of nuclei with DAPI (blue channel). Two set are shown (S2, S3). The same exposure was used to take images in the cases of the WT and *orb2* mutants. The neuropile region are marked with green line. The areas marked by the yellow line are the neuronal body regions. Scale bar, 100 µm.

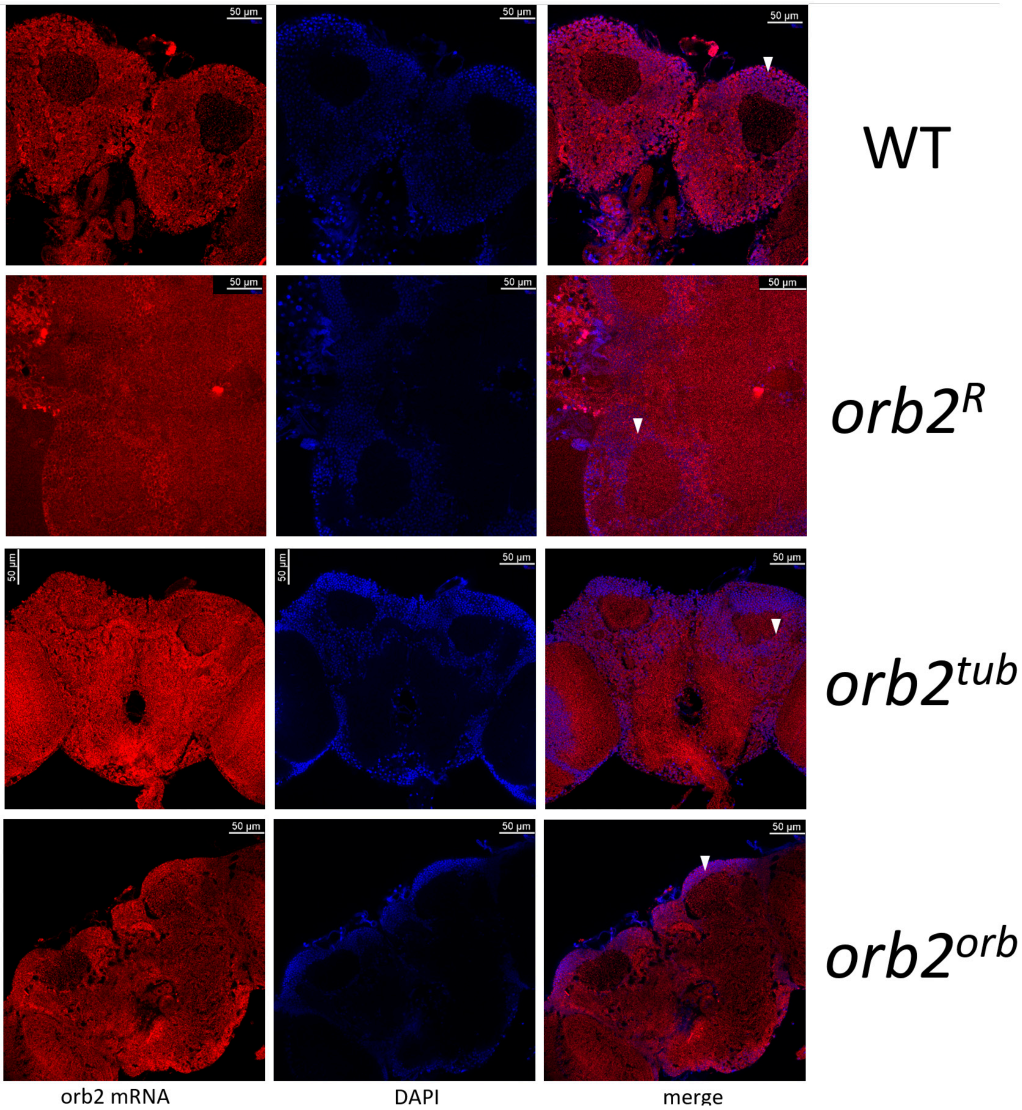

Figure S4.

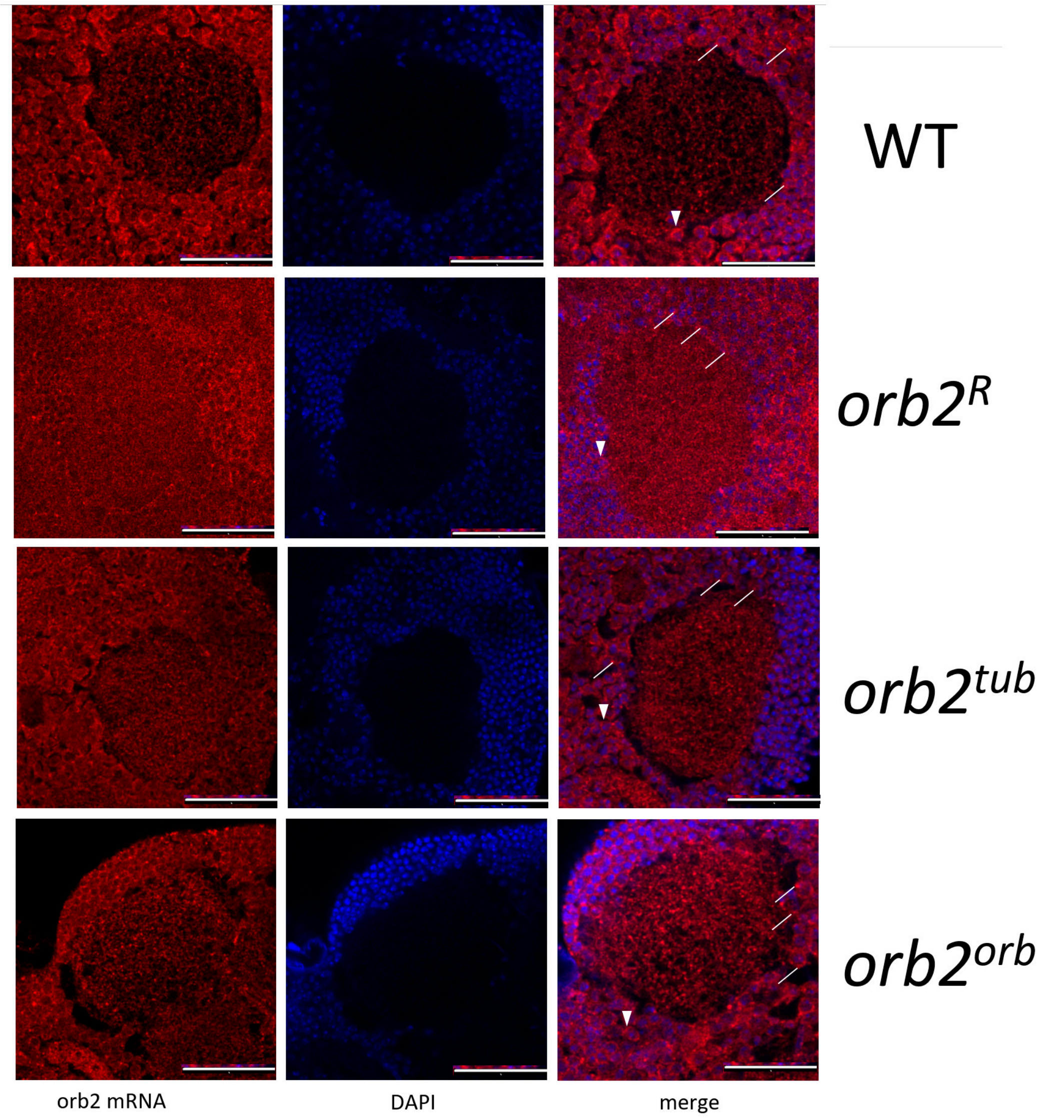

Figure S5. Brain preparations of the WT, *orb2^R*, *orb2^{tub}*, and *orb2^{orb}* lines. Overall view (S4) and enlarged calyx region (S5). Red channel, staining of whole mount brain samples with anti-*orb2* probes; blue channel, staining of nuclei with DAPI. White triangles indicate the perinuclear ring of *orb2* mRNA in the neuronal soma. White lines indicate bright specks of *orb2* mRNA near the neuronal nucleus. Figure S4, scale bar, 50 μm; Figure S5, scale bar, 5 μm.

Primers used in study:

gapdh2_forward: 5′-CTACCTGTTCAAGTTCGATTCGAC-3′

gapdh2_reverse: 5′-AGTGGACTCCACGATGTATTCG-3′

act5C_forward: 5′-GGCACCACACCTTCTACAATGAGC-3′

act5C_reverse: 5′-GAGGCGTACAGCGAGAGCACAG-3′

orb2_forward: 5′-TAACACCAGCGAAAGGGGAC-3′

orb2_reverse: 5′-TCAGATGTGCGACGAGTGC-3′

28S RNA_forward: 5′-AATTCAGAACTGGCACGGACTTGG-3′

28S RNA_reverse: 5′-AGAGCACTGGGCAGAAATCACATTG-3′

Csp_forward: 5′-ATAGCCAAGCCGCGAAGCCA-3′

Csp_reverse: 5′-CGGGCTAAGCCGCAAGGGAT-3′

Pyd_forward: 5′-CGGAACGCCGAGCAGCAATG-3′

Pyd_reverse: 5′-AGCCATTCGGAGAACGGGGC-3′

Tubulin cloning_forward: 5′-ATGACTCATCGTCCCAGAGA-3′

Tubulin cloning_reverse: 5′-TTGCCAGCTCACCGAAAT-3′

Orb cloning_forward 5′-GGATCCACTGTTACGGCTTTTATC-3

Orb cloning_reverse 5′-AGATCTGAATTCAATAGCGATTTTG-3′

Sequence of αTub84B 3′UTR

GCGTCACGCCACTTCAACGCTCGATGGGAGCGTCATTGGTGGGCGGGGTAAC-CGTCGAAATCAGTGTTTACGCTTCCAATCGCAACAAAAAATTCACTGCAACACTGAAAAGCATACGAAAACGATGAAGATTGTACGAGAAACCATAAAGTATTTTATCCACAAAGACACGTATAGCAGAAAA-GCCAAGTTAACTCGGCGATAAGTTGTGTACACAAGAATAAAATCGGCCAGATTCAGTGTTGTCAGAAATAAG

Sequence of orb 3′UTR:

ACTGTTACGGCTTTTATCCACACCGTTTTAACGGATGTTTCCGCAATATAATGTGTGA-GACTTTGGACTTGTAGGCGACGTAGAAATATGGCGGAGATAGTTTTCTTGCAGCCGATGGAGACCCGGGCTCATCATCGTCATTATCATTGTAAACACAGTTTTATTGATGTGTATATAATATCGGTACAA-TATGAAGCTTACACTTTGAGTTTCATTTAAGATAATTATTGTTAGACGACTCCCCCAAAAACGAACACCCTCGAATCGAAACAGAAGAGAGACCCCCATTCATACTATTTTTTGATAATCATGCTTACATATCAG-CATTTGGAGCTGGCATTCGAATGCTAAATGAATGATACATGAAATGAGCAATTTCATTTACTCATACCCCTCATACAAATACTACTCAAGTTACTTTTTGGCTAAGAAAGCAGTTATTAATTTCTAATTT-GTATGTTTAATTTTAAGGAAACGAAACCGCGCGGCAAATGGCTACTTGAAATGTCTCGACCAATTTGCCGCGCTGCGAGATTAGAAACACCATTTTTGTTATGTTTTGTGCTATTACTGAGGAATTTTATGTAG-TTTCTTTTTACATAGCCAAGCCCCCGACTCGAGTTAATATGATATTATATTATTTGGATTGTCCGCTAAGCGTTTATCAGGAATTTCAATTTTTAAGAAAACATTTTAAAAATTGTAAATTCGTTTAACTCACCAG-TCTCCCTTTTTTGTTTTTCATTGAATTGTTACACACATGAGTTATTATAAATATACTGTTTAGTTTATTTTTTGTTTTGGATTAGCTTTAAGCATTATCCTTGTGAACATTAACGCGATGCCTGATTGATTGTTGAAC-TATGTTTTAAGCATTTATCATTCTTTGGCTTTTTTGGCTGTTCCAGTTTTATAGCTCATGGGCAATAAGCATATAATTTATGTCTACTTTATTTTTCATGTATTTTGAGAAAGGATCTCTCTAGCGCTTATCTATA-GAAAACACACATATGTATGTATACAAAAATATTACATAGTGTACATTACAAGGCTTTATATAATTTAAAACTTGATAAGTTTGTAAACCTACACAGAATAGGAAAAAAATACTTAAGTCTATATCTTAAACAC-GAATTGATCACTAAACGATAAAATAACGCACACACACCTATTCACCAACCAAACAAATATACAAAATCGCTATTGAATTC

Sequences of STELLARIS probes for orb2 mRNA.

5′- CGTCAGCAACGACTGTGATG CGTTGTTTTCTTTCCTGATT CTTTCCGTACAACGGTAACT AATTCCTTT-GCACTCGATTC GGTGCACACTCACACAAACG CAGTGTGCGTGTTACATTTC TTTCAGGTGCAAGTTTTTCC GAGAGCGCACGAAGATGGAA ACCAACACTTTCTACAGCTG CTTTGGTAACTTGAGCGAGT CTGAC-GATGTAGGCGATGTG CATTGAATCGCACCGAGATC CTATGCTTGCTGTGATGGAA CAGACTAACTTCGTCGTTGC CACCGGCTTGCATGAAATTA CACCGCAACTTTTTCGATTG GTTGAGATTGA-GATTCGGCA TGGGAAAGGATATGCTGCGA CATGTGCATATTGCCATAGA TATATTCATGTGCCCACTAC CACCGTTTCCAATCGAAAGT CATAGCTGTTGCCCAAATAG AATCGCACAGTGAGTTATCC GTCCTG-CATTAAAGACATGC TAGACGGATGGATCATTTCC AATCTCATCCTCATCGATGT CGAAGCGCCGAAAC-GAAGTG CCTTGGGCGGAAAATACGAC TGGAACAGCAGGAAGGCATA CACGAGTCAATTAGCTGCTG CGTCGGCGAAGAAACGCATA CAAGGACGAATCTGCACTGC CATGGTAGCATCAAGCACAT CACAAACAC-CGTTTTGCGTG CACCGTACAATCTATCCATG GTCAATTCCAGCATAGCATA GCCCTTTGGATACTTTAATT AGCTCTGCTGATTCGAGAAG ACAAATCTGGCTGAGATGGC GCGCAAAAGAAGGGCGCAAA AGTGTTCG-CAATAGTACTGC TAACACCAGCGAAAGGGGAC TTTAACAACCGGTGTCGTTG TGTTATGATTCTGGGCACTG TATCTACTAGATTCTTCGCC TGGTGGCTATTCAAAGGGAC TCGTTTCTCGCCTCTAAAAC TGCAGGCATCGAACGCAAAA-3′
